# The effect of enhancers on the lentiviral transduction efficiency in the human RPE cells: Insights for advancing retinal gene therapies

**DOI:** 10.1016/j.bbrep.2025.102010

**Published:** 2025-04-14

**Authors:** Sajad Najafi, Azam Rahimpour, Hamid Ahmadieh, Mozhgan Rezaei Kanavi, Maryam Maleki Tehrani, Fatemeh Suri, Javad Ranjbari

**Affiliations:** a Department of Medical Biotechnology, School of Advanced Technologies in Medicine, Shahid Beheshti University of Medical Sciences, Tehran, Iran; b Cellular and Molecular Biology Research Center, Shahid Beheshti University of Medical Sciences, Tehran, Iran; c Ophthalmic Research Center, Research Institute for Ophthalmology and Vision Science, Shahid Beheshti University of Medical Sciences, Tehran, Iran; d Medical Nanotechnology and Tissue Engineering Research Center, Shahid Beheshti University of Medical Sciences, Tehran, Iran; e Department of Tissue Engineering and Applied Cell Sciences, School of Advanced Technologies in Medicine, Shahid Beheshti University of Medical Sciences, Tehran, Iran; f Ocular Tissue Engineering Research Center, Research Institute for Ophthalmology and Vision Science, Shahid Beheshti University of Medical Sciences, Tehran, Iran

**Keywords:** Lentivirus, Retina, RPE, Transduction, Polybrene, Protamine sulfate

## Abstract

**Background:**

Viral vectors including lentiviruses (LV), adenoviruses (AV) and adeno-associated viruses (AAV) have been used as common vehicles for gene transfer in gene therapy of various human diseases. The efficacy of gene transfer, however, still remains unsatisfying and thus, a number of biologic and chemical substances are used for enhancing the transduction efficiency. In this article, we aim to evaluate the cytotoxicity and impact of individual and combinational treatment of two polycationic agents hexadimethrine bromide (polybrene; Pb) and protamine sulfate (PS) on the transduction efficiency of lentiviral particles in the primary human retinal pigment epithelial (RPE) cells.

**Methods:**

Cytotoxicity of Pb and PS at individual and combinational concentrations was evaluated using MTT cell viability assay in RPE cells. Lentiviral particles were produced using a set of second-generation vectors and different combinations of two enhancers, Pb and PS, were added to the transduction medium. The transduction efficiency of lentiviral particles in RPE cells was evaluated using flow cytometry and calculating the mean fluorescence intensity (MFI), as well as the percentage of green fluorescent protein (GFP)-positive cells. All the treatments were performed in three replicates.

**Results:**

Cell viability assay revealed that individual treatment of Pb at all concentrations by up to 25 μg/ml was safe to RPE cells with no visible toxicity and its combination with PS did not significantly improve its effect on the cell viability. Interestingly, Pb at all concentrations significantly improved the transduction efficiency compared to control virus with the best MFI result seen at 10 μg/ml concentration. The mean population of GFP-positive cells was also most enhanced at that concentration (p-value: 0.006). At a combinational concentration of 10 μg/ml of Pb and 2 μg/ml of PS, the highest level of transduction efficiency was reported (MFI: 801, GFP+: 65.4 %); however, the value was not significant when compared to enhancers used in individual treatments or relative to other combinations.

**Conclusion:**

Pb enhanced the transduction efficiency of lentiviral particles in RPE cells and in combination with PS achieved the highest level of MFI and GFP-positive percentage. Although, the efficiency of the combination was not significant compared to that of individual treatments, this study may suggest the potential of combinational enhancers for applications in gene therapy.

## Highlights

• Polybrene (Pb) at conventional concentrations did not demonstrate any evidence of cytotoxicity in human retinal pigment epithelium (RPE) cells.
• The addition of Pb at all concentrations resulted in a significant enhancement in lentiviral transduction efficiency.
• The 10 μg/ml concentration of Pb demonstrated the most optimal transduction efficiency.
• The combination of Pb and protamine sulfate (10 μg/ml and 2 μg/ml, respectively) demonstrated the highest level of transduction efficiency.

## 1 Introduction

The inherited retinal diseases (IRDs) constitute a group of heterogenous genetic eye diseases that are characterized by progressive vision impairment and blindness [1]. More than 280 causative genes are identified in association with IRDs (Retinal Information Network, https://sph.uth.edu/retnet/), while a majority shows an autosomal recessive inheritance [2,3]. Gene therapy as a promising approach for the treatment of IRDs has received increasing attention during the last decades. It is partly due to several features making eye as a good target for gene therapies. Structurally, the eye is a small, accessible and compartmentalized organ which allows local administration of low volumes of biological agents by direct visualization without use of invasive protocols. Local administration and the immune privilege provided by the blood-ocular barrier decrease the side effects and immunogenic systemic responses compared to the systemic administration being applied for the gene therapy of some other organs [4]. Advances in the development and application of viral vectors have made the clinical translation of ocular gene therapies a reality. Delivery approaches mainly determine the success of gene therapy. Compared to non-viral vectors, viral vectors have robust transduction efficiency and thus, are more frequently used as the vehicles for transferring a gene of interest into the target cells [5]. These vectors are based on viral backbones that are naturally developed to infect some specific types of cells. Adeno-associated viruses (AAVs), adenoviruses (AVs), herpes simplex viruses (HSVs), retroviruses, and lentiviruses (LVs) are the main viral-based vectors most widely studied in gene therapy preclinical experiments and clinical trials [6]. Advances in vector engineering, delivery, and safety during the past two decades have led to the introduction of several viral vector-based gene therapy products for clinical administration in various monogenic diseases [7,8]. According to the American depository of clinical trials (www.clinicaltrials.gov), among a handful of experiments of gene therapies for various IRDs, at least two studies (NCT06388200 and NCT04850118) are recruiting cases in the phase III of gene therapies for treating retinitis pigmentosa (RP). Notably, voretigene neparvovec‐rzyl (Luxturna) was a recombinant AAV-based gene therapy product that received regulatory approval from the United States Food and Drug Administration (FDA) for the treatment of a specific type of retinal dystrophy associated with biallelic mutation in the *retinal pigment epithelium-specific 65 kDa* (*RPE65*) gene [9].

Although significant progresses have been achieved, gene delivery approaches still have limited success rates due to several challenges like inefficient cell uptake and low transduction efficiency [10,11]. To improve the efficiency of gene delivery, a number of materials are used particularly for affecting the transfection and transduction success [10]. These include polymers, such as polyethyleneimine (PEI), peptides, lipid formulations like lipofectamine, nanoparticles, including calcium phosphate (Ca_3_(PO_4_)_2_), and drugs (e.g., camptothecin). 1,5-Dimethyl-1,5-diazaundecamethylene polymethobromide (hexadimethrine bromide; also known as polybrene) is a cationic polymer that has been widely used since 1990s for enhancing the efficiency of gene transfer by viral vectors [12,13]. It acts through decreasing the charge-repulsion between the vector and the surface of the target cell [14]. Polybrene is a cost-effective and accessible agent that has adequate safety measures and simple handling making it a popular additive for enhancing transduction of several types of viral vectors including LVs [15]. However, polybrene shows toxicity particularly for *in vivo* application and thus, some combinations have been suggested to overcome this limitation [16]. The cytotoxicity of polybrene is particularly visible when the concentration is increased, causing cell membrane disruption [17]. Polybrene; however, is still among the most commonly used additives in gene therapy and thus, employing this polycation at less concentrations with preserved gene delivery efficacy in combination with other agents may be suggested as an alternative strategy when compared to its individual application. Protamine sulfate is another additive composed of polycationic peptides originally extracted from salmon sperm, which is used for improving transduction efficiency and possesses less toxicity when compared to polybrene [10]. In several studies, protamine sulfate has shown an enhancing impact on the transduction efficiency of LVs similar to that seen for Vectofusin-1 [18] and the efficiency is even further improved in combinational treatment [19]. However, it is less studied compared to other agents particularly in retinal gene delivery. The current study aims to evaluate the effect of individual and combined polybrene and protamine sulfate treatments on the cell viability and the lentiviral transduction efficiency in primary human retinal pigment epithelial (RPE) cells.

## 2 Methods

This study was approved by the Research Ethics Committees of Vice-Chancellor in Research Affairs, Shahid Beheshti University of Medical Sciences, Tehran, Iran (ethical approval code: IR.SBMU.RETECH.REC.1401.368). The posterior eyecups of donated eyes were used for primary human RPE cell culture based on the informed consents of the Central Eye Bank of Iran (Tehran, Iran) and in accordance with the declaration of Helsinki regulations.

### 2.1 Cells and culture

The human embryonic kidney (HEK) 293T and Lenti-X 293T cells were purchased from the Iranian Biological Resource Center. Cell culture was conducted in Dulbecco's modified Eagle Medium (DMEM; Idea Zist, Iran) supplemented with 10 % fetal bovine serum (FBS; Gibco, United States) and 1 % penicillin/streptomycin (Pen-Strep, Gibco). The cells were plated in T75 flasks (SPL Life Sciences, South Korea) and incubated at 37 °C in incubator supplied with 5 % CO_2_.

Primary human RPE cells were isolated from three human donor posterior eyecups provided by the Central Eye Bank of Iran and cultured as previously reported [20]. The adonors were aged 24, 29, and 35 years and represented both sexes. The cases were subjects of confirmed brain death secondary to suicide or trauma. Following the collection of eyeballs, they were transferred on ice to the laboratory up to 5 h post-collection. Briefly, the human posterior eyecups were radially cut into four quadrants. After removal of the sensory retina and the vitreous, the RPE/choroidal layer was separated from the sclera, cut into small segments, and transferred to a Petri dish containing DMEM and 1 mg/ml of dispase I (Invitrogen, Brussels, Belgium). The mixture was incubated in 37 °C with 5 % CO_2_ for 90 min, centrifuged at 300 g and 4 °C for 5 min, and transferred to a T25 flask with 20 % FBS-enriched DMEM. RPE cells were grown after 3–7 days and then checked for morphology and confluence for eventual passaging in T75 flasks. For enzymatic digestion, cells were washed with sterile PBS and then treated with trypsin-EDTA (Idea Zist, Iran) for up to 5 min, inactivated by adding 10 % FBS-supplemented DMEM and then the cell pellet was harvested by centrifuging at 2700 rpm for 5 min. Identification of RPE cells was routinely conducted using two antibodies, the goat polyclonal anti-human RPE65 and goat polyclonal anti-human Cytokeratin 8/18 (1:1000; Santa Cruz Biotechnology, Dallas TX, USA) (data not shown). No variability was seen in the behaviour among three expanded RPE cells from different sources.

### 2.2 Vectors and transfection

The lentiviral backbone plasmid (pCDH-CMV-MCS-EF1-cGFP-T2A-puro; Plasmid #72263**,** Adgene, Cambridge, MA) and the second-generation LV packaging vectors (pMD2G and psPAX2; Plasmids #12259, and #12260, Adgene, Cambridge, MA) were received as gifts from Dr. Arefian at the Molecular Virology Laboratory, Department of Microbiology, School of Biology, College of Science, University of Tehran, Tehran, Iran, and used for the LV production. All vectors were extracted using Maxi-Prep plasmid extraction columns according to the manufacturer's instructions (#FAPDE000, Favorgen, Taiwan). The concentration of the extracted plasmid DNA was measured using a Nanodrop 2000C spectrophotometer (Thermo Scientific, USA) and then confirmed by gel electrophoresis. The PEI linear polymer of 25 KDa molecular weight (Polysciences Inc., Pennsylvania, USA) at an adjusted pH of 7.0 was used for the transfection of Lenti-X 293T cells. The transfected cells were then incubated overnight, the medium was changed with fresh 10 % FBS-supplemented DMEM on the next day. The green fluorescent protein (GFP) expression was investigated 24–72 h post-transfection using fluorescence microscopy (Olympus IX71, Tokyo, Japan) equipped with a digital camera (Olympus U-TV0.63XC; Tokyo, Japan).

### 2.3 Lentivirus packaging and infection

Fresh Lenti-X 293T cells were seeded in T75 flasks at 70 % confluence, and passages between 3 and 15 were used for lentiviral packaging. One day later, PEI-mediated co-transfection of host cells was conducted using three vectors. According to the protocol of viral particle production, the supernatant was collected at multiple times, including 48-, 60-, and 72-h post-transfection, filtered using a 0.45μm filter, and then concentrated by adding Lenti-X Concentrator (Takara Bio, Kusatsu, Japan), overnight incubation, and eventually centrifugation (according to the manufacturer's instructions). The viral titer and multiplicity of infection (MOI) were then calculated in HEK-293T cells. A fixed MOI value was used for the transduction of different RPE cells (from distinct sources) and across experiments. This value was optimized by examining various values used in RPE cells compared to the basic value evaluated in HEK-293 cells.

### 2.4 Enhancers

Polybrene was purchased from Bonyakhteh, Iran, and protamine sulfate was provided from Merck, Germany. Both enhancers were used 1 h prior to the viral treatment of RPE cells. To evaluate the impact of the combined polybrene-protamine sulfate on the lentiviral transduction efficiency in RPE cells, different concentrations of both enhancers were examined (Table 1). Based on the literature, a concentration range of 6 to 10 μg/ml was used for polybrene and protamine sulfate treatment was conducted at 2–6 μg/ml concentrations [[21], [22], [23]]. 72 h post-transduction, treated RPE cells were evaluated by flow cytometry to calculate the percentage of GFP-positive cells as an indicator of transduction efficiency. To avoid any possible bias occurring in a specific extracted population of primary human RPE cells, treatment was repeated on two other RPE sources extracted from distinct human posterior eyecups (triplicate treatments).

**Table 1:** The design of combinational treatment of polybrene and protamine sulfate in lentivirus-added RPE cells. To evaluate the effect of both enhancers, including single treatments and combined administration for investigating the possible synergism, different concentrations of polybrene (6-, 8-, and 10 μg/ml) and protamine sulfate (2-, 4-, and 6 μg/ml) were used for the treatment of RPE cells.

	Sample	Polybrene (μg/ml)	Protamine sulfate (μg/ml)
1	Control cell (no virus, no enhancer)	–	–
2	Virus control (virus+, no enhancer)	–	–
3	0/2	–	2
4	0/4	–	4
5	0/6	–	6
6	6/0	6	–
7	8/0	8	–
8	10/0	10	–
9	6/2	6	2
10	6/4	6	4
11	6/6	6	6
12	8/2	8	2
13	8/4	8	4
14	8/6	8	6
15	10/2	10	2
16	10/4	10	4
17	10/6	10	6

### 2.5 MTT cell viability assay

To assess the possible cytotoxic impact of enhancers on RPE cells, the 3-(4, 5-dimethylthiazol-2-yl)-2, 5-diphenyltetrazolium bromide (MTT) reduction assay was used for the investigation of cell viability based on the instructions from the substance and reagents’ manufacturer (Sigma Aldrich, Darmstadt, Germany). Briefly, in a 96-well plate a number of 10,000 RPE cells were seeded in each well at day 0 and treated with enhancers at day 1. At day 2, 100 μl of MTT solution was added to each well, incubated at 37 C for 4 h and then replaced with an equal volume of DMSO. The optical density (OD) of cells at wells was measured at 540 nm and 630 nm-wavelengths using an Elx808 automated microplate photometer (BioTec Instruments, Inc., USA). For estimation, absorbance at both wavelengths was subtracted and then cell viability was calculated according to the following formula.

$$Viability\;\left(\%\right)=\frac{\left(OD_{sample}-Blank\;absorbance\right)}{\left(Mean\;OD_{control}-Blank\;absorbance\right)}\times 100$$

### 2.6 Flow cytometry

A total number of 2.0 × 10^5^ RPE cells were seeded in a 24-well plate. Next day, the medium was changed with fresh DMEM enriched with 10 % FBS and the cells were incubated with enhancers and the concentrated LV solution. 48 h post-transduction, the cells were twice washed with PBS and then detached with trypsin-EDTA. FBS-supplemented DMEM was added for trypsin inactivation, and the cell pellet was harvested by centrifuging. The percentage of GFP-positive RPE cells was calculated by flow cytometry as follows.

$$GFP‐positive\;RPE\;cells\;\left(\%\right)=\frac{Number\;of\;GFP‐positive\;cells}{Number\;of\;GFP‐positive\;cells+number\;of\;GFP‐negative\;cells}\times 100$$

Non-transduced RPE cells were used as the negative control sample for setting the fluorescence threshold and accordingly, the background fluorescence (autofluorescence) was excluded ensuring that only GFP-positive cells were selected. The gating strategy was based on SSC-H (Side Scatter Height) versus FL1-H (Fluorescence Channel 1 Height, for GFP) plot for the separation of GFP-expressing cells from the background noise. The mean fluorescence intensity (MFI) was also quantified for each well to compare the mean fluorescence power for presenting a comparable parameter of transduction efficiency along with GFP-positive percentage.

### 2.7 Statistical analysis

Graphpad Prism 8.0 (GraphPad Software, USA) was used for data analysis. Comparison of values between groups was conducted using one-way ANOVA followed by Tukey test. A ∗p value of <0.05 was considered statistically significant. To ensure repeatability, experiments were conducted in triplicate.

## 3 Results

### 3.1 Cytotoxicity assay

**Fig. 1:**
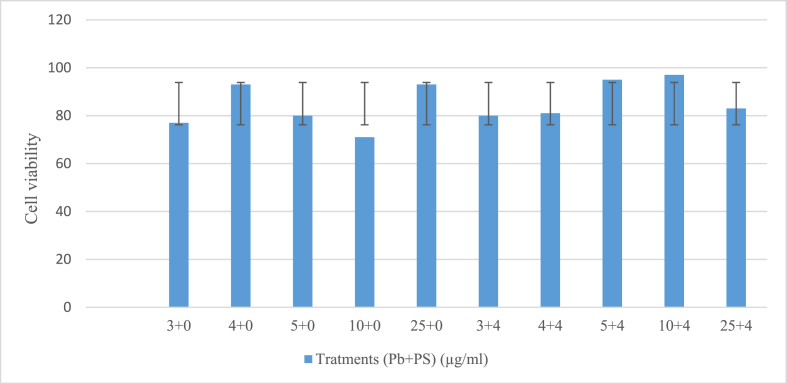
MTT cell viability assay for polybrene (Pb) and protamine sulfate (PS) concentrations in RPE cells. Primarily, upon treatment of cells with an individual dose of Pb (3-, 4-, 5-, 10-, and 25 μg/ml), by increasing the concentration, no significant dose-dependent cytotoxicity was seen (p-value> 0.05), revealing that Pb did not significantly impact the viability of RPE cells. The combinational treatment of Pb and PS also had no effect on cell viability compared to untreated control and individual Pb-treated cells. Each experiment was conducted for three times.

Human RPE cells were successfully isolated and identified from three distinct human donors. Those cells were used for three reactions of evaluating cytotoxicity and transduction efficiency. MTT cell viability assay revealed that polybrene at all concentrations by up to 25 μg/ml did not show any significant cytotoxicity compared to control RPE cells with no enhancer treatment (p-values> 0.05) (Fig. 1). The combined treatments also had no significant change in cytotoxicity relative to polybrene individual treatment. Based on the findings in MTT assay, we confirmed that those concentrations of both enhancers are safe to RPE cells and then evaluated their impact on the lentiviral transduction efficiency.

### 3.2 Transduction efficiency

**Fig. 2:**
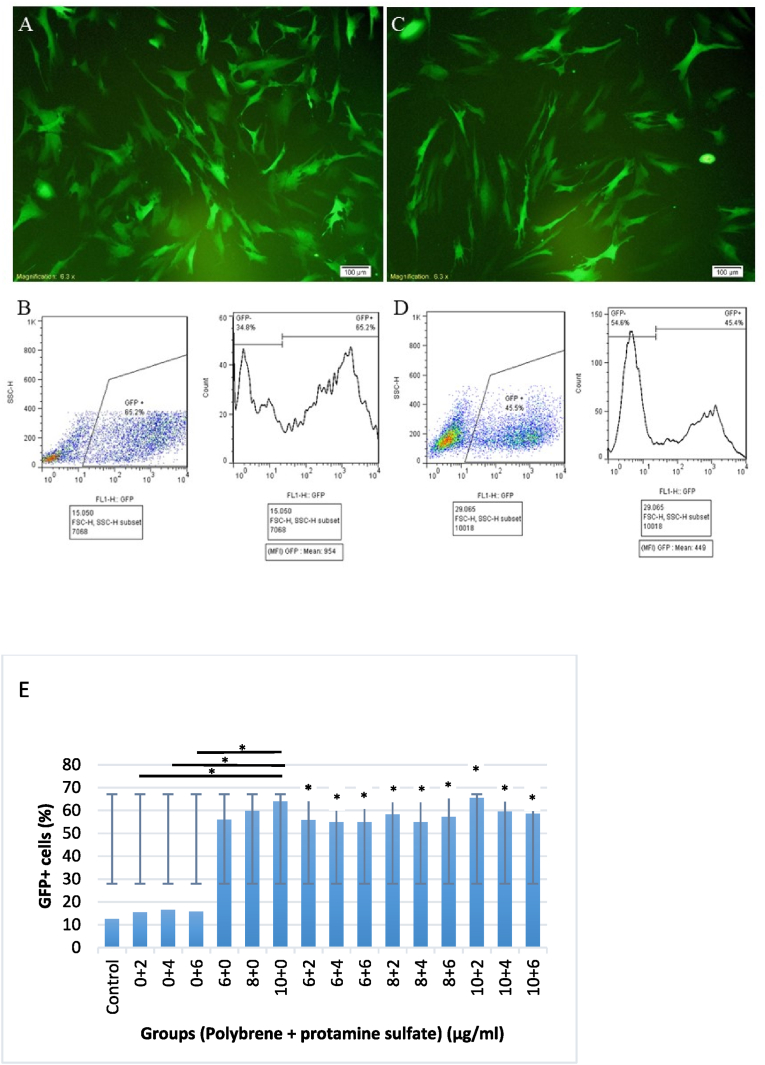
The impact of polybrene and protamine sulfate on the lentiviral transduction efficiency in RPE cells using flow cytometry. Data are shown for GFP + percentage of transduced RPE cells. Polybrene showed an enhancing impact on the transduction efficiency in various concentrations compared to individual virus transduction with no added enhancer. (**A and B**) demonstrate fluorescence micrograph and flowcytometry graph, respectively for a combined concentration of 10 μg/ml polybrene and 2 μg/ml of protamine sulfate; (**C and D**) show the corresponding documents of fluorescence micrography and flowcytometry graph, respectively for polybrene at 8 μg/ml. **(E)** The graph illustrates the comparison of GFP + population of RPE cells in flowcytometry for all concentrations. The control column is indicative of transduced RPE cells with no enhancer treatment. Each experiment was conducted for three times. The symbol ∗ illustrates the significance difference (p-value> 0.05) among the transduction efficiency of various treatments compared to virus particles individually used for the treatment of RPE cells. It also shows a significant improvement of transduction efficiency for polybrene at 10 μg/ml concentration compared to protamine sulfate at three concentrations.

To evaluate the possible effect of polybrene and protamine sulfate on the transduction efficiency of LVs in RPE cells, serial concentrations of both enhancers were examined on three human eye-expanded RPE cell sources. RPE cells were seeded on three distinct 24-well plates and enhancers were added to lentiviral treatment. The results of fluorescence microscopy demonstrated successful transduction of RPE cells that were then confirmed using flow cytometry and investigation of GFP expression. Comparing the percentage of GFPexpressing cells and MFI revealed some differences among the lentiviral transduction efficiency affected by the various concentrations of both enhancers. Accordingly, adding polybrene individual significantly improved the transduction efficiency of LVs that was shown by significant enhancement of the mean population of GFP + cells as compared to that of control virus-treated cells (56 %, 59.86 %, and 63.93 % for 6-, 8-, and 10 μg/ml concentrations, respectively; p-values: 0.04, 0.01, and 0.006, respectively) (Fig. 2, E). Notably, polybrene at a concentration of 10 μg/ml demonstrated the highest efficiency on transduction improvement that was higher than two other concentrations (GFP + percentage of 63.93 %, and MFI of 679 vs those values of 59.86 % and 555 for 8 μg/ml, as well as 56 % and 549 for 6 μg/ml, respectively); however, the values were not significantly different (p-values> 0.05). On the other side, when protamine sulfate was used individual, it did not show any significant enhancement of lentiviral transduction relative to the control viral treatment with no additive (p-values> 0.999). At a concentration of 4 μg/ml, protamine sulfate showed the best effect on the lentiviral transduction but the values did not change significantly when compared to no-additive control virus treatment (mean GFP+: 16.5 %, p-value> 0.999). Of note, adding combined polybrene and protamine sulfate (10 μg/ml and 2 μg/ml, respectively) achieved the best result by reaching an MFI of 801.66 and mean GFP+ of 65.4 %.

## 4 Discussion

Although vigorous advances have been made in developing gene delivery vehicles, the transduction efficiency of viral vectors still requires improvement. Polybrene is a polycationic agent considered as a conventional additive with approved function has been used for enhancing viral transduction efficiency in gene therapies [21]. It is a cost-effective agent and offers simple and safe handling, and thus, polybrene has been used as a promoter in virus-mediated gene therapy trials and research experiments on various viruses like Ads and LVs to various cell recipients, such as mesenchymal stem cells (MSCs) and immune cells [24,25]. In ocular cells the conventional treatment with polybrene has been used *in vitro* for facilitating the transduction efficiency of viral vectors into keratocytes [26], human umbilical vein endothelial cells (HUVECs) [27], retinal stem cells (as an acoustic wave-mediated gene delivery) [28], and RPE cells [29]. Polybrene, particularly in higher concentrations, causes toxicity like neural degeneration and cochlear hair cell necrosis, which is the major limitation for its *in vivo* application [30,31]. Overcoming the current cytotoxicity of polybrene may suggest it for broader applications in gene delivery. A potential strategy can be through combinational treatments through which the administered dose of polybrene does not express negative impact on the transduction efficiency. Theoretically, this effect may be contributed to yielding an optimum transduction efficiency at a low-dose, safe concentration of polybrene upon combining with another enhancer possessing a synergistic or additive impact on transduction. The findings of our study are comparable to the intraocular injection of polybrene at a final concentration of 8 μg/ml *in vivo* [29], and we show that polybrene obviously enhances the transduction efficiency in the human RPE cells *in vitro* by a dose-dependent manner. Protamine sulfate is another polycationic agents with applications for enhancing gene transfer efficiency with a long history of implications in gene therapies [32,33]. To the best of our knowledge, protamine sulfate has not been studied in retinal gene delivery studies, and we for the first time examined its impact on the transduction efficiency of LVs. Polybrene showed a significant impact on promoting the transduction efficiency compared to individual virus-treated cells, while it was safe to RPE cells by up to 25 μg/ml concentration. The effect of polybrene was consistent with previous studies and supports its long-term use in trials and research experiments. The impact of protamine sulfate was not significantly higher than control virus-treated cells. Of note, although a small increase was seen, the combination of polybrene and protamine sulfate failed to reflect a synergistic yield of transduced cells and also had no significant impact on the viability of RPE cells compared to individual polybrene-treated cells. This data may show no efficiency of protamine sulfate for viral transduction at least in RPE cells. Polybrene in an individual treatment can significantly improve the percentage of cells receiving the transgene and accordingly, can benefit gene therapy approaches to overcome the delivery limitation. Although polybrene showed no toxicity in RPE cells by a total concentration of 25 μg/ml, an efficient transduction was seen for lower concentrations (optimum at 10 μg/ml) that are conventionally considered as non-toxic [17]. Higher concentrations of polybrene were not examined in the current study, while in the previous studies it is reported with a concentration-dependent cytotoxicity against other cells [31,34,35]. Although, the precise mechanism(s) responsible for the absence of a synergistic impact is not elucidated, some potential explanations include the saturation of receptors and charge interactions. However, there is no known interaction between these enhancers through which they may offer a novel gene therapy treatment. Exploring other agents with different modes of action and also optimizing dosage ratios may be helpful. These findings can be employed for designing a combinational additive regimen for the maximum enhancing of the viral transduction efficiency with applicability for *in vivo* treatments. Although MTT assay in the current study revealed the safety of both enhancers at their conventional dosages, finding potential off-target effects and extending the lack of toxicity beyond 72 h post-transduction can simulate the real biological environment. Additionally, larger studies to investigate a wider range of concentrations for both enhancers may find a more efficient combination. Investigation of higher concentrations of polybrene for *in vitro* and *in vivo* applications are also necessary. Particularly, the components of extracellular matrix (ECM) may impact the effectiveness of cationic polymers, possibly through affecting their distribution. Thus, clinical administration needs considering the possible impact of ECM contents on the enhancer distribution and effectiveness. Exploring the synergistic impact of other polycationic agents for finding optimized combinational treatments is also suggested. *In vivo* experiments and studies beyond the organotypic cultures may properly address the potential application of either individual or combinational administration of two substances for an improved gene delivery approach.

## Authorship contribution statement

Javad Ranjbari and Fatemeh Suri conceptualized the study, Sajad Najafi implemented the study and wrote the primary manuscript, Azam Rahimpour, Hamid Ahamdieh, Mozhgan Rezaei Kanavi, and Maryam Maleki Tehrani contributed to the methodology and interpretation, Fatemeh Suri and Azam Rahimpour revised the manuscript, Fatemeh Suri and Javad Ranjbari supervised the study and finalized the manuscript. All authors contributed to and have read the submitted manuscript.

## Ethics approval

The study was approved by Research Ethics Committees of Vice-Chancellor in Research Affairs, Shahid Beheshti University of Medical Sciences (ethical approval code: IR.SBMU.RETECH.REC.1401.368).

## Availability of data and material

The data used for the study will be available upon a reasonable request to the corresponding authors.

## Consent for publication

Authors of this paper have read the final version of the manuscript and approve to submit the paper in the journal.

## Funding

This study was supported by Shahid Beheshti University of Medical Sciences (Grant No: 30958/IR.SBMU.RETECH.REC.1401.368).

## Declaration of competing interest

The authors declare that they have no known competing financial interests or personal relationships that could have appeared to influence the work reported in this paper.

## Data Availability

Data will be made available on request.
